# Commentary: Metformin reverses TRAP1 mutation-associated alterations in mitochondrial function in Parkinson's disease

**DOI:** 10.3389/fnagi.2018.00221

**Published:** 2018-07-31

**Authors:** Sachchida N. Rai, Saumitra S. Singh, Hareram Birla, Walia Zahra, Aaina S. Rathore, Payal Singh, Surya P. Singh

**Affiliations:** ^1^Department of Biochemistry, Institute of Science, Banaras Hindu University, Varanasi, India; ^2^Department of Biochemistry, Institute of Medical Science, Banaras Hindu University, Varanasi, India

**Keywords:** Parkinson disease, mitochondria, metformin, TRAP1, HtrA2, PINK1

Mitochondrial quality is a very prominent factor for cell homeostasis. Recently, it has been noticed that the primary focus in Parkinson's disease (PD) pathogenesis has been on mitochondrial dysfunction and its underlying pathway. Tumor necrosis factor type 1 receptor (TRAP1) is a chaperone responsible for maintaining the mitochondrial quality and energy metabolism found in the mitochondrial matrix (Altieri et al., 2012; Rasola et al., 2014). The function of TRAP1 is severely impaired in Parkinsonism, in both humans and animals, and hence it may be a potential target in PD pathogenesis. TRAP1 acts as the stress sensor responsible for helping the cells to adjust to the changes in environment, and is phosphorylated by Parkinson's disease kinase (PINK1) (Plun-Favreau et al., 2007; Pridgeon et al., 2007). HTRA2 is the protein having activity similar to that of TRAP1, and wherein both are phosphorylated by PINK1. Researchers have shown that in both humans and animals, the loss of function of PINK1 along with HTRA2 causes Parkinsonism. However, overexpression of TRAP1 rescues this loss of function, hence making it clear that TRAP1 acts downstream of HTRA2 and PINK1 (Fitzgerald et al., 2017).

In this paper, by using mice as an animal model, Fitzgerald et al. have shown that TRAP1 is a very prominent protein responsible for maintaining the energy homeostasis along with the quality of mitochondria in PD. Fitzgerald et al. have also suggested that TRAP1 interacts non-canonically with HTRA2 to maintain the homeostasis, by using an unbiased mass spectrometric approach (Fitzgerald et al., 2017). TRAP1 is an indirect target of HTRA2 as it potentially regulates the protein level of TRAP1. Fitzgerald et al. have clearly shown that in late-onset Parkinsonism, the first TRAP1 mutation causes total loss of functional protein as compared to healthy control. Moreover, the level of stress and oxygen consumption have increased, and the total ATP and quantity of mitochondria have decreased in PD patients. These findings in PD patients completely diminished the mitochondrial biogenesis along with the quality of mitochondria.

To confirm that HTRA2 physically interacts with TRAP1, Fitzgerald et al have used the technique of unbiased mass spectrometry. They have also performed immunoprecipitation by using HeLa cells, which suggest that both are regulated by each other. Moreover, Fitzgerald et al have also demonstrated that TRAP1 effectively rescues loss-of-function of HTRA2 and PINK1, however, TRAP1 is not a proteolytic substrate of HTRA2 (Fitzgerald et al., 2017).

Glucose metabolism and diabetes-related complications have been relieved by the widely-used drug metformin, although the mechanism involved is not yet fully investigated. Glucose production in the liver has been shown to be reduced by metformin, though its mechanism of action is still to be fully understood. It has been noticed that the gut might have a key role to play in its mechanistic action. With the obvious difference between acute and chronic administration, the findings at the molecular level may vary depending on the doses of metformin used and the duration of treatment. Metformin action involves both AMP-activated protein kinase (AMPK)-dependent and AMPK-independent mechanisms which inhibit mitochondrial respiration, and which could also cause inhibition of mitochondrial glycerophosphate dehydrogenase. Lysosomes can also play an important role in Metformin mediated inhibition (Rena et al., 2017). Metformin efficiently rescues the reduced mitochondrial membrane potential and shows neuroprotective activity in PD, as demonstrated earlier (Patil et al., 2014; Pérez-Revuelta et al., 2014). It is reported that among patients with diabetes mellitus type 2, who consume metformin, there are fewer cases of PD (Wahlqvist et al., 2012). In this published paper, Fitzgerald et al also have found that metformin, a diabetes drug, rescues the loss created by TRAP1 mutation and restores mitochondrial biogenesis, along with mitochondrial membrane potential, by using *in vitro* and *in vivo* along with the knockout model. Consequently, in PD, the detection of TRAP1 as a novel HTRA2 interactor encourages exploration of the PINK1-HTRA2-TRAP1 pathway (Fitzgerald et al., 2017). However, it is necessary to validate the role of TRAP1 in familial PD and also in sporadic PD.

Development of PD in diabetic patients has been strongly supported by reports published in esteemed journals. The long-term use of metformin in diabetic patients shows the beneficial effect of improving motor dysfunction as compared to other anti-diabetic drugs. The mechanism responsible for metformin's beneficial activity is not fully known. However, researchers have suggested that activation of AMPK-dependent pathways in human neural stem cells might be responsible for the neuroprotective activity of metformin. Amelioration of oxidative damage by metformin has been clearly suggested by numerous *in vitro* and *in vivo* studies. The effect of metformin on non-motor symptoms is not well-known. Therefore, there is a strong need to investigate the effect of metformin on the gut-microbiome axis as it is from here that the non-motor symptom is initiated.

To summarize, Fitzgerald et al. have shown that for the proper functioning of mitochondria, TRAP1 acts downstream to PINK1 and HTRA2, while its loss of function leads to improper control of energy metabolism affecting mitochondrial membrane potential. Their findings allow us to understand the pathobiology of mitochondria in PD and can act as a target for medicinal therapy. Overall, this interesting study opens the window to search for another therapeutic compound which can show activity similar to that of metformin.

## Author contributions

All authors listed have made a substantial, direct and intellectual contribution to the work, and approved it for publication.

### Conflict of interest statement

The authors declare that the research was conducted in the absence of any commercial or financial relationships that could be construed as a potential conflict of interest.
